# A stepwise multi-disciplinary algorithm for diagnosis of fibrosing lung diseases contributing MDCT, MRI, and PET/CT: a study on 250 patients using significance and validation analyses

**DOI:** 10.1186/s43055-022-00928-4

**Published:** 2022-11-22

**Authors:** Ahmed Samir, Mohamed Hossameldin Khalifa, Ayman Ibrahim Baess, Rania Ahmed Sweed, Ahmed Mohamed Abougabal, Aya Abdel Galeel

**Affiliations:** 1 Department of Radio-Diagnosis, Faculty of Medicine, Alexandria University, Alexandria, Egypt; 2 Department of Chest Diseases, Faculty of Medicine, Alexandria University, Alexandria, Egypt

**Keywords:** Algorithm, Fibrosing lung diseases, CT, MRI, PET/CT

## Abstract

**Background:**

The new guidelines limited the use of lung biopsy in the evaluation of lung fibrosis because of its hazards. The differential diagnosis of interstitial pulmonary fibrosis (IPF) or usual interstitial pneumonia (UIP) is challenging because of overlapping multi-detector computed tomography (MDCT) morphologic features between interstitial and non-interstitial fibrosing lung diseases. Scar carcinoma is a serious complication that needs to be excluded in certain conditions. *Aim of the work:* To achieve a multi-disciplinary algorithm for the diagnosis of fibrosing lung diseases to limit the need for lung biopsy by combining the clinico-laboratory and radiological roles.

**Results:**

This study included two major steps. The first step (prevalence/significance analysis of the contributing parameters for the diagnosis of fibrosing lung diseases) was retrospectively conducted on 150 patients pathologically proved with fibrosing lung disease during the period between January/2016 and April/2018. Based on a *P*-value < 0.001, honeycombing bronchiectasis was significant to IPF. Basal traction bronchiectasis/bronchiolectasis was relevant to fibrosing non-specific interstitial pneumonia (NSIP). "Head cheese" CT-sign, history of allergen exposure, blood eosinophilia, and broncho-alveolar lavage (BAL) lymphocytosis were relevant to chronic hypersensitivity pneumonitis (HP). Upper peripheral lung fibrosis was significant to pulmonary tuberculosis (TB) and pleuroparenchymal fibroelastosis (PPFE). Cavitations, tree-in-bud, and calcific nodules were relevant to TB, while the "platy-thorax" CT-sign was relevant to PPFE. The upper peribronchovascular fibrosis was relevant to sarcoidosis and progressive massive fibrosis (PMF); additionally, calcific changes were relevant to PMF. Bright T2-signal, diffusion weighted-image (DWI) restriction in magnetic-resonance imaging (MRI), and high standardized uptake value (SUV) in positron emission tomography (PET-CT) were significant to scar carcinoma. Eventually, an algorithm was created. The second step (validation analysis) prospectively targeted 100 patients initially diagnosed with lung fibrosis during the period from June/2018 to June/2022. It revealed 83.3–100% sensitivity, 96.3–100% specificity, 85.7–100% PPV, 96.4–100% NPV, and 96–100% accuracy, with balanced accuracy = 0.91–1. Four consulting radiologists and two consulting pulmonologists participated in this study.

**Conclusions:**

A valid stepwise multi-disciplinary algorithm was proposed for the diagnosis of interstitial and non-interstitial fibrosing lung diseases to limit the need and hazards of lung biopsy. It contributed significant clinico-laboratory data, MDCT features, T2-WI and DWI-MRI findings as well as PET/CT results.

## Background

In the past, the role of chest imaging was restricted to diagnosing lung fibrosis and estimating its extent. Currently, the MDCT plays a major role in the characterization of idiopathic interstitial pneumonias (IIPs) and the differentiation between these interstitial and other non-interstitial fibrosing lung diseases [1, 2]. Controlling some of these diseases could eventually prevent further progression of lung fibrosis to improve the quality of life and increase the five-year survival rate [3].

The updated classification of chronic fibrosing IIPs differentiated the usual interstitial pneumonia (UIP) or interstitial pulmonary fibrosis (IPF) as well as the pleuroparenchymal fibroelastosis (PPFE) from the other non-UIP types of IIPs such as fibrosing non-specific interstitial pneumonia (fibrosing NSIP) and chronic hypersensitivity pneumonitis (HP) [4].

UIP is the most common type of IIPs which is characterized by irreversible interstitial fibrosis and honeycombing bronchiectasis with poor response to therapy and poor prognosis. Pleuroparenchymal fibroelastosis is a rare type of IIP which is characterized by upper lobar rapidly progressive peripheral fibrosis and also poor prognosis [5].

Fibrosing NSIP is characterized by basal ground glass changes and interstitial thickening with traction bronchiectasis; meanwhile, chronic HP is caused by repetitive exposure to a certain allergen and characterized by mixed ground glass changes and air trapping with interlobular septal thickening. On the contrary, these non-UIP diseases could profit from the use of corticosteroids and new anti-fibrinogenic drugs with better outcomes [6, 7].

Other non-interstitial causes of lung fibrosis included pulmonary tuberculosis (TB), sarcoidosis, progressive massive fibrosis (PMF), and post-irradiation fibrosing pneumonitis. Fibro-thorax is a serious complication of pulmonary tuberculosis with fibro-atelectasis, calcifications, and parenchymal distortion [8]. Sarcoidosis is a systemic inflammatory disease that commonly affects middle age females with skin erythema and arthralgia and results in upper lobar peri-bronchial fibrosis with parenchymal distortion and bronchiectatic changes [9]. Progressive massive fibrosis is a result of pneumoconiosis with high mortality that commonly distorts the lobular lung parenchyma with calcific changes and architectural distortion [10].

Prolonged fibrosis and scarring are risk factors for secondary malignancy "scar carcinoma," mostly in heavy smoker patients with TB [11].

Pulmonary fibrosis expresses low T2-WI signal intensity. On the other hand, the high T2-WI signal intensity and diffusion restriction are considered strong predictors for malignant changes [12]. PET-CT can confirm malignant changes through hot uptake and high SUV [13].

Because of the overlapping morphologic CT features, the collaboration between the clinical and radiological roles could minimize the need for lung biopsy and avoid unnecessary invasive complications [3].

*Aim of the work:* To achieve a multi-disciplinary algorithm for the diagnosis of interstitial and non-interstitial fibrosing lung diseases that limit the need for lung biopsy, combining the clinico-laboratory data with the MDCT findings, the T2-WI and DWI-MRI characteristics, and PET/CT results.

## Methods

**Fig. 1:**
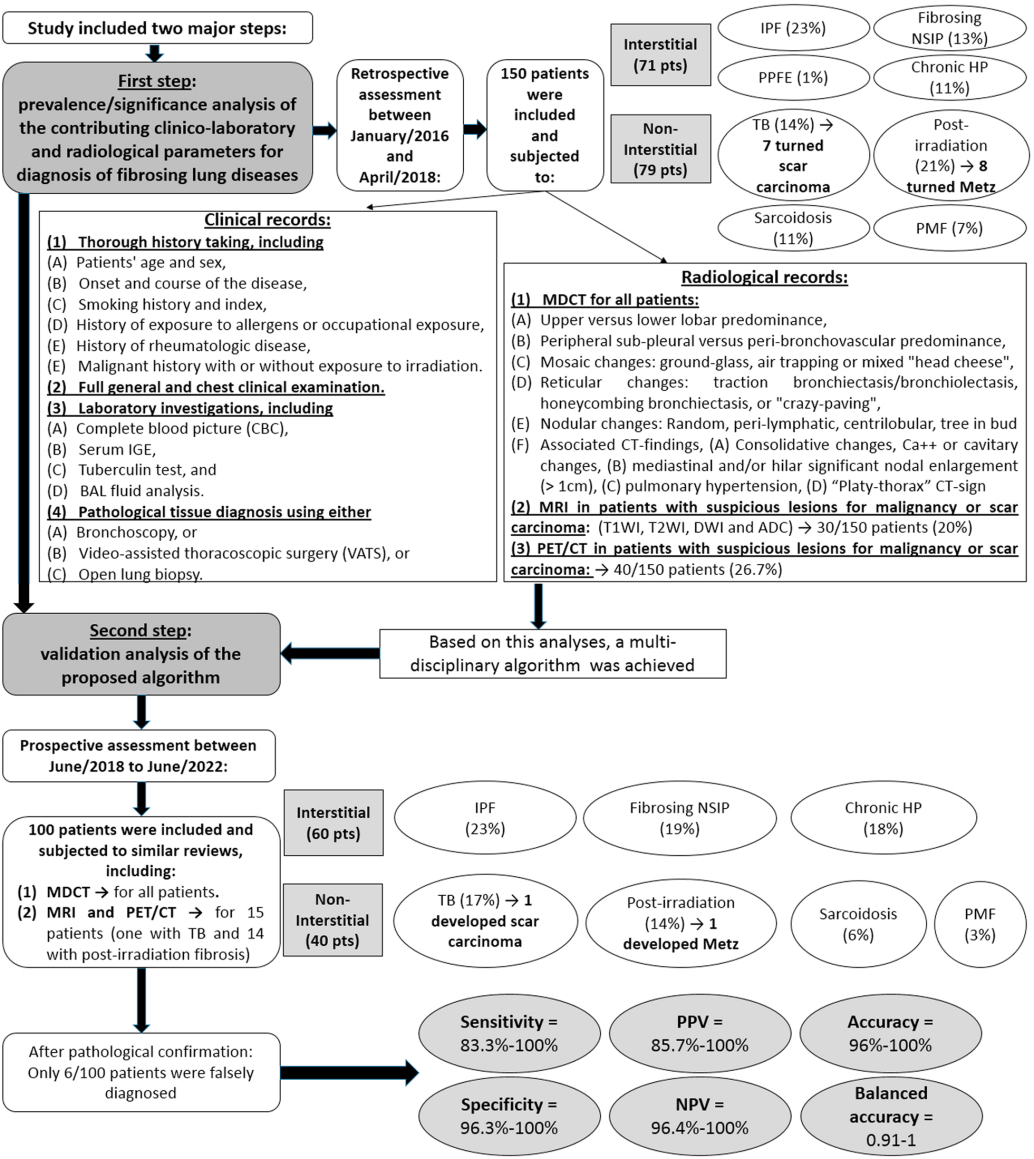
A flow-diagram is demonstrating the study design and methodological steps with brief results

A flow-diagram is demonstrating the study design and methodological steps with brief results (Fig. 1).

This study included two major steps: the first step was a prevalence/significance analysis of the contributing clinico-laboratory and radiological parameters for the diagnosis of fibrosing lung diseases to create a stepwise multi-disciplinary algorithm, while the second step was a validation analysis regarding this proposed algorithm.

The study had received approval from the "Institutional Ethics Committee." The "Research Ethics Board" had waived patient consent after assuring full respect for the confidentiality of the patient's data and their medical records. This manuscript does not overlap with any previously published works.

Overall, four expert consulting radiologists and two consulting pulmonologists participated in this study; the radiological experience ranged from 10 to 25 years, while the clinical experience averaged 16 and 20 years.

### First step (prevalence/significance analysis to create an algorithm)

It was retrospectively conducted on 150 patients pathologically proved with fibrosing lung disease during the period between January/2016 and April/2018. It tested the prevalence and significance of the contributing clinico-laboratory and radiological factors for the diagnosis of fibrosing lung diseases. Using the statistically proven significant parameters, a multi-disciplinary algorithm was created.

Inclusion criteria were as follows: (1) Patients diagnosed with either interstitial or non-interstitial fibrosing lung disease with complete medical records and positive pathological results using either (A) bronchoscopy with guided tissue biopsy or broncho-alveolar lavage (BAL), or (B) video-assisted thoracoscopic surgery (VATS) with tissue biopsy, or (C) open lung biopsy. (2) Patients with full radiological workup including MDCT with MRI and/or PET/CT in patients with suspicious lesions for malignancy or scar carcinoma.

Exclusion criteria were as follows: (1) patients with incomplete medical records, and (2) patients with a degraded quality of radiological images.

*Clinical records***:** Two consulting pulmonologists participated in this step with clinical experience averaging 16 and 20 years. All patients were subjected to: (1) Thorough history taking, including (A) patients' age and sex, (B) onset and course of the disease, (C) smoking history and index, (D) history of exposure to allergens or occupational exposure to industrial dust, (E) History of rheumatologic diseases, and (F) malignant history with or without exposure to irradiation. (2) Full general and chest clinical examination.

*Laboratory investigations* included either (A) complete blood picture (CBC), (B) serum IgE, (C) tuberculin test, or (D) BAL fluid analysis.

*Pathological diagnosis* included either (A) bronchoscopy, (B) video-assisted thoracoscopic surgery (VATS), or (C) open lung biopsy.

*Radiological records*

All patients were subjected to MDCT examinations. Intravenous contrast administration was only used in MDCT examination of patients with lymphadenopathy or malignancy.

Multiple MDCT machines were utilized as follows: (1) GE LightSpeed Plus 4 slice CT scanner (USA), (2) Canon Medical Systems; Toshiba Aquilion 64, Japan, (3) Canon Medical Systems; Toshiba Aquilion CXL/CX 128, Japan, and (4) SOMATOM Sensation 64, Siemens Medical Systems, Germany,

MDCT scanning parameters were as follows: (1) Slice thickness was 1 mm for 64 and 128-slices MDCT and 2.5 mm for 4-slices MDCT, (2) tube rotation was 0.6 s for 64 and 128-slices MDCT and 0.9 s for 4-slices MDCT, (3) Detector Collimation was 1 mm, (4) kVp ranged from 80–120, and (5) mA ranged from 120–240.

MDCT characteristics were carefully studied, including (1) upper versus lower lobar predominance, (2) peripheral sub-pleural versus peri-bronchovascular predominance, (3) mosaic changes such as ground-glass (GG) attenuation, air trapping, or mixed ("head cheese" sign), (4) reticular changes such as traction bronchiectasis/bronchiolectasis, honeycombing bronchiectasis, or "crazy-paving" appearance made from smooth interlobular septal thickening on top of GG attenuation, (5) nodular changes either (A) centrilobular with or without a tree-in-bud pattern, (B) axial or peripheral peri-lymphatic spread, or (C) Randomly located nodules with or without calcifications, (6) associated CT-findings, including (A) consolidative changes with or without calcifications or cavitary changes, (B) mediastinal and/or hilar significant nodal enlargement (> 1 cm in short axis diameter), (C) pulmonary hypertension (pulmonary trunk > 3.3 cm in caliber or exceeding caliber of the nearby ascending thoracic aorta), and (D) the "platy-thorax" CT-sign which means flattening of the anterior chest wall with decreased anteroposterior diameter.

MRI examinations were conducted using a 1.5-Tesla scanner (GE Medical Systems, Sigma-Aldrich, USA). Each study included axial T1-weighted (TR/TE, 550/15), axial and coronal T2 FSE (3000/120), and DWI (b-value 0, 500, and 1000) with ADC mapping using single-shot echo-planar spin-echo sequences.

PET/CT examinations were conducted using a hybrid PET/CT scanner (Siemens Biograph 64 PET/CT scanner). The usual precautions and preparation were respected as follows: (1) Six hours before the examinations, patients were instructed for fasting except for water and to avoid heavy muscular exercises to avoid false positive results of muscular over-uptake. (2) Patients were instructed for urine voiding before the examination. (3) At the time of examinations, blood glucose level was less than 150 mg/dl, and patients were instructed to stop talking. Around 5MBg/kg body weight of 18-FDG was injected. High SUV (> 3) is considered a predictor of malignancy.

In the first step, two expert consulting radiologists analyzed the radiological findings in a consensus; with radiological experience averaging 10 and 18 years. They were informed of all available relevant data.

Statistical analysis was as follows: (1) Prevalence of each type of fibrosing lung disease was calculated, (2) prevalence of each clinical or laboratory or pathological or radiological contributing parameters was calculated, and (3) statistical analysis of the significant relationship between each of these contributing parameter and final diagnosis. Chi-square tests and *P*-value measurements were estimated using an online calculator (https://www.socscistatistics.com). *P*-value (< 0.05) was considered statistically significant.

### Second step (validation analysis for the proposed algorithm)

It prospectively targeted patients with suspected lung fibrosis during the period from June/2018 to June/2022 who were referred from the outpatient clinic for chest diseases.

Patients proved with post-COVID fibrosis were initially excluded.

All referred patients were subjected to an initial MDCT examination to exclude patients without CT signs of lung fibrosis. Eventually, 100 patients with CT signs of lung fibrosis were included in this step of the study.

These finally included 100 patients who were subjected to the application of the proposed algorithm through similar full medical and radiological assessments.

MRI and PET/CT examinations were routinely performed in patients with irradiation pneumonitis (14 patients) and furtherly needed in a single patient with suspected scar carcinoma on top of post-TB fibrosis. The final diagnosis was confirmed pathologically.

Other two expert consulting radiologists participated in the second step, with radiological experience averaging from 11 and 25 years. They worked together in a consensus. The same two consulting pulmonologists participated in this step.

Statistical analysis of validation was as follows: Prevalence, sensitivity, specificity, positive predictive value (PPV), negative predictive value (NPV), accuracy, and balanced accuracy were calculated using an online diagnostic test calculator (https://www.medcalc.org/calc/diagnostic_test.php).

## Results

### First step (prevalence/significance analysis)

Table 1 summarizes the first step results.

**Table 1:** Comparison between UIP/IPF and other fibrosing lung diseases with prevalence and significance analyses

		Interstitial lung diseases							
Diseases		UIP/IPF		Fibrosing NSIP		Chronic HP		PPFE	
Number of patients and percentage:		34 (23%)		19 (13%)		16 (11%)		2 (1%)	
Number/percentage and *P*-value		*N*/%	*P*-value*	*N*/%	*P*-value	*N*/%	*P*-value	*N*/%	*P*-value
**MDCT characteristics**									
* Lobar distribution	Upper lobe predominance	–	–	–	–	–	–	2(100)	0.17
	Lower lobe predominance	34(100)	**< 0.001**	19(100)	**< 0.001**	16(100)	**< 0.001**	–	–
* Axial distribution	Peripheral/sub-pleural	34(100)	**0.007**	19(100)	0.06	8(50)	**< 0.001**	2(100)	0.57
	Peri-bronchovascular	–	–	12(63)	0.08	16(100)	**< 0.001**	–	–
* Mosaic pattern	Ground glass opacities (GGOs)	34(100)	**< 0.001**	19(100)	**< 0.001**	16(100)	**< 0.001**	–	–
	Mosaic perfusion (Air trapping)	11(32)	**0.004**	11(58)	0.72	16(100)	**< 0.001**	–	–
	Ground glass versus air trapping predominance	GG		GG		Almost equal		Absent	
	Head cheese mixed pattern.	–	–	3(16)	0.66	16(100)	**< 0.001**	–	–
* Reticular pattern	Traction bronchiectasis and bronchiolectasis	34(100)	**< 0.001**	19(100)	**0.001**	5(31)	**< 0.001**	–	–
	Honeycombing	34(100)	**< 0.001**	–	–	–	–	–	–
	Crazy paving pattern	–	–	–	–	16(100)	**< 0.001**	–	–
* Nodular pattern	Centrilobular nodules	–	–	–	–	6(38)	**< 0.001**	–	–
	Peri-lymphatic nodules	–	–	–	–	–	–	–	–
	Calcific random nodules	–	–	–	–	–	–	–	–
* Associated signs	Consolidation and calcifications	–	–	–	–	–	–	2(100)	**0.035**
	Cavitation	–	–	–	–	–	–	–	–
	L.Ns (> 1cm short axis)	–	–	–	–	–	–	–	–
	Pulmonary hypertension	15(44)	**< 0.001**	7(37)	**0.049**	4(25)	0.6	–	–
	Platy-throrax	–	–	–	–	–	–	2(100)	**< 0.001**
**MRI characteristics**									
* T2WI	Hypo-intense signal	N/A	N/A	N/A	N/A	N/A	N/A	N/A	N/A
* DWI	No restriction (high ADC)	N/A	N/A	N/A	N/A	N/A	N/A	N/A	N/A
**History**									
* Exposure	Certain allergen/Birds Fancier	–	–	–	–	13(81)	**< 0.001**	–	–
	Occupational exposure	–	–	–	–	–	–	–	–
	Malignancy/irradiation	–	–	–	–	–	–	–	–
* Past history	*Rheumatologic*	–	–	15(79%)	**< 0.001**	–	–	–	–
**Lab results**									
* CBC and IgE	Eosinophilia	–	–	–	–	12 (75)	**< 0.001**	–	–
* BAL	Lymphocytosis	N/A	N/A	N/A	N/A	12 (75)	**< 0.001**	N/A	N/A
* Tuberculin T.	Positive	N/A	N/A	N/A	N/A	N/A	N/A	–	–
* Nodal biopsy	Bronchoscopic guided	N/A	N/A	N/A	N/A	N/A	N/A	N/A	N/A
**Malignant sequel (Scar carcinoma or Metz)**									
* Incidence, prevalence and significance		–	–	–	–	–	–	–	–
* T2WI	Hyper-intense signal.	N/A	N/A	N/A	N/A	N/A	N/A	N/A	N/A
* DWI	Restriction (low ADC)	N/A	N/A	N/A	N/A	N/A	N/A	N/A	N/A
* PET/CT	High FDG uptake and high SUV:	N/A	N/A	N/A	N/A	N/A	N/A	N/A	N/A
* Biopsy	VTAS: Metaplasia/neoplasia	N/A	N/A	N/A	N/A	N/A	N/A	N/A	N/A

		Non-interstitial fibrosing lung diseases							
Diseases		sarcoid		PMF		Post-TB		Post-irradiation	
Number of patients and percentage:		16 (11%)		11 (7%)		21 (14%)		31 (21%)	
Number/percentage and *P*-value		*N*/%	*P*-value	*N*/%	*P*-value	*N*/%	*P*-value	*N*/%	*P*-value
**MDCT characteristics**									
* Lobar distribution	Upper lobe predominance	16(100)	**< 0.001**	11(100)	**< 0.001**	21(100)	**< 0.001**	27(87)	**< 0.001**
	Lower lobe predominance	–	–	–	–	–	–	4(13)	**< 0.001**
* Axial distribution	Peripheral/sub-pleural	5(31)	**< 0.001**	9(82)	0.678	21(100)	**0.046**	31(100)	**< 0.001**
	Peri-bronchovascular	16(100)	**< 0.001**	11(100)	**< 0.001**	12(57)	0.215	–	–
* Mosaic pattern	Ground glass opacities (GGOs)	3(19)	**< 0.001**	–	–	–	–	27(87)	**< 0.001**
	Mosaic perfusion (Air trapping)	3(19)	**0.018**	11(100)	**0.001**	21(100)	**< 0.001**	8(26)	**< 0.001**
	Ground glass versus air trapping predominance	GG>air trapping		Sub-pleural emphysema		Sub-pleural emphysema		Sub-pleural GG	
	Head cheese mixed pattern.	–	–	–	–	–	–	–	–
* Reticular pattern	Traction bronchiectasis and bronchiolectasis	6(38)	**0.006**	5(45)	**0.01**	18(86)	**< 0.001**	15(48)	**< 0.001**
	Honeycombing	–	–	–	–	–	–	–	–
	Crazy paving pattern	–	–	–	–	–	–	16(52)	**< 0.001**
* Nodular pattern	Centrilobular nodules	5(31)	**< 0.001**	–	–	–	–	–	–
	Peri-lymphatic nodules	16(100)	**< 0.001**	–	–	–	–	–	–
	Calcific random nodules	–	–	5(45)	0.051	21(100)	**< 0.001**	7(23)	0.93
* Associated signs	Consolidation and calcifications	14(88)	**< 0.001**	11(100)	**< 0.001**	20(98)	**< 0.001**	–	–
	Cavitation	–	–	–	–	5(24)	**< 0.001**	–	–
	L.Ns (> 1cm short axis)	15(94)	**< 0.001**	1(9)	0.058	21(100)	**< 0.001**	16(52)	**0.02**
	Pulmonary hypertension	4(24)	0.6	–	–	–	–	–	–
	Platy-throrax	–	–	–	–	–	–	–	–
**MRI characteristics**									
* T2WI	Hypo-intense signal	N/A	N/A	N/A	N/A	21(100)	**< 0.001**	31(100)	**< 0.001**
* DWI	No restriction (high ADC)	N/A	N/A	N/A	N/A	15(71)	**< 0.001**	23(74)	**< 0.001**
**History**									
* Exposure	Certain allergen/Birds Fancier	–	–	–	–	–	–	–	–
	Occupational exposure	–	–	11(100)	**< 0.001**	–	–	–	–
	Malignancy/irradiation	–	–	–	–	–	–	31(100)	**< 0.001**
* Past history	*Rheumatologic*	–	–	–	–	–	–	–	–
**Lab results**									
* CBC and IgE	Eosinophilia	–	–	–	–	–	–	–	–
* BAL	Lymphocytosis	N/A	N/A	N/A	N/A	N/A	N/A	N/A	N/A
* Tuberculin T.	Positive	–	–	–	–	5(24)	**< 0.001**	N/A	N/A
* Nodal biopsy	Bronchoscopic guided	15(94)	**< 0.001**	N/A	N/A	N/A	N/A	N/A	N/A
**Malignant sequel (Scar carcinoma or Metz)**									
* Incidence, prevalence and significance		–	–	–	–	7/21(33)	**< 0.001**	8/31(26)	**< 0.001**
* T2WI	Hyper-intense signal.	N/A	N/A	N/A	N/A	7/7(100)	**< 0.001**	8/8(100)	**< 0.001**
* DWI	Restriction (low ADC)	N/A	N/A	N/A	N/A	7/7(100)	**< 0.001**	8/8(100)	**< 0.001**
* PET/CT	High FDG uptake and high SUV:	N/A	N/A	N/A	N/A	7/7(100)	**< 0.001**	8/8(100)	**< 0.001**
* Biopsy	VTAS: Metaplasia/neoplasia	N/A	N/A	N/A	N/A	7/7(100)	**< 0.001**	8/8(100)	**< 0.001**

*P-value < 0.05 is considered significant. *N/A: Not applicable

1. The type and complications of fibrosing lung disease

Non-interstitial fibrosing lung diseases were encountered in 79/150 patients (52.7%), including post-irradiation pneumonitis (31/150–21%), post-TB fibrosis (21/150–14%), sarcoid disease (16/150–11%), and progressive massive fibrosis on top of pneumoconiosis (11/150–7%).

Chronic fibrosing IIPs were less common (71/150–47.3%), including UIP/IPF (34/150–23%), fibrosing NSIP (19/150–13%), chronic hyper-sensitivity pneumonitis (16/150–11%), and PPFE (2/150–1%).

Malignancy only complicated non-interstitial fibrosing lung diseases. It was detected in 15/150 patients (10%) and proved by MRI and PET-CT. Eight patients had metastatic spread on top of post-irradiation changes. Seven patients were pathologically proved with scar carcinoma that complicated post-TB fibrosis.

2. The clinical records and laboratory results

The included patients were 92 males and 58 females (61.3%:38.7%). Their age ranged from 33 to 76 years (mean age 58.4 ± 11.3 SD)**.**

The disease showed an insidious course in 145/150 patients (96.7%); meanwhile, two patients (0.7%) with PPFE and three patients (2%) with post-irradiation pneumonitis and fibrosis had rapidly progressive dyspnea.

Smoking history was positive in 48/150 patients (32%). It was relevant to pulmonary TB (76.2% prevalence and < 0.001 *P*-value). It was also significant to scar carcinoma (100% prevalence and < 0.001 *P*-value).

History of exposure to certain allergens was positive in 13 female patients who raise different kinds of birds (bird-fancier) and proved with chronic hypersensitivity pneumonitis (81% prevalence and < 0.001 *P*-value).

History of rheumatologic diseases was relevant to chronic fibrosing NSIP (79% prevalence and < 0.001 *P*-value).

Skin erythema and arthralgia were relevant signs of sarcoid disease (75% prevalence and *P*-value < 0.001, each).

The blood eosinophilia and BAL lymphocytosis were relevant to chronic hypersensitivity pneumonitis (75% prevalence and *P*-value < 0.001, each).

3. The radiological findings

Involvement of the upper lung lobes was relevant to non-interstitial fibrosing lung disease (*P*-value < 0.001). It was encountered in 87% of patients proved with post-irradiation fibrosis and 100% of patients proved with pulmonary TB, sarcoidosis, and PMF. On the other hand, lower lobar involvement was relevant to chronic fibrosing IIPs including UIP, fibrosing NSIP, and chronic HP (100% prevalence and *P*-value < 0.001).

The peri-bronchovascular localization of the fibrosing process was significant to chronic HP, sarcoidosis, and PMF (100% prevalence and *P*-value < 0.001).

**Fig. 2:**
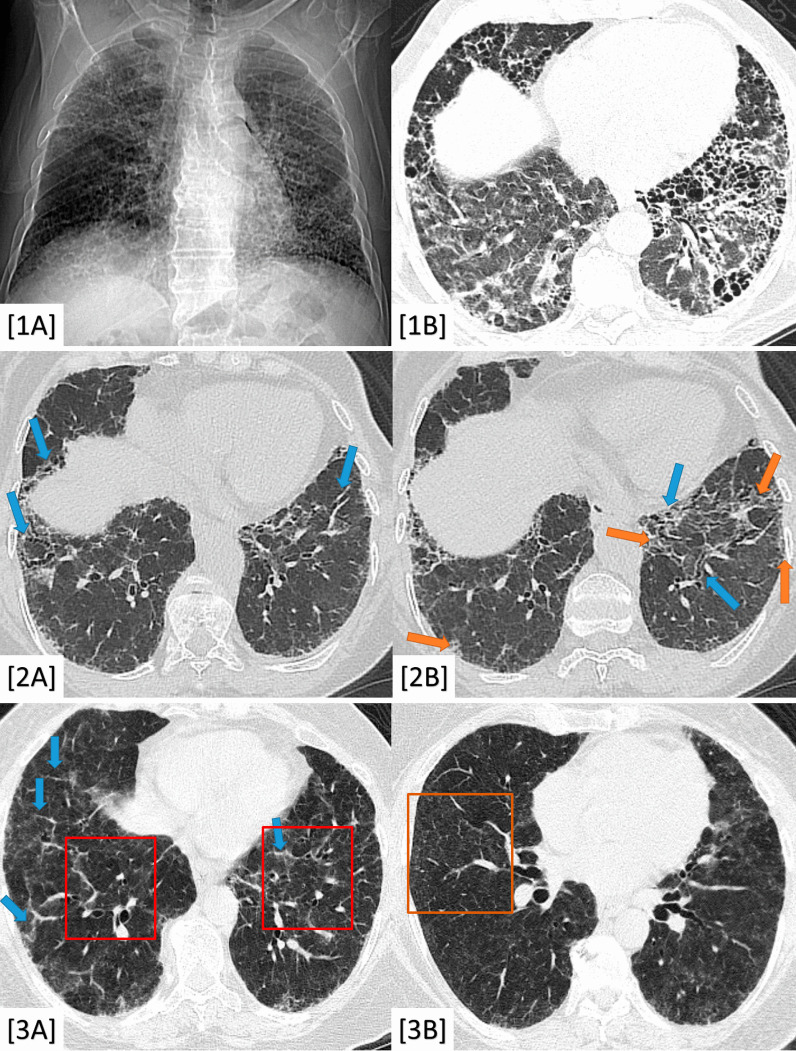
1**A**, **B** A 55-year-old male patient complaining of progressive dyspnea with restrictive pulmonary functions; 1**A** X-ray chest PA view, 1**B** Axial chest CT (lung window); both showing extensive fibrotic changes and honeycombing bronchiectasis, more pronounced in lower lung zones. Diagnosis: Proved patient with UIP/IPF. 2**A**, **B** A 53-year-old female patient with a history of rheumatoid arthritis who was complaining of progressive dyspnea (2**A**, **B**) Axial chest CT (lung window); both showing bilateral lower lobar mosaic ground-glass attenuation (orange colored arrows) mixed with peri-bronchial thickening and traction bronchiectasis/bronchiolectasis (blue colored arrows) without honeycombing. Diagnosis: Proved patient with fibrosing NSIP. 3**A**, **B** A 43-year-old female patient complaining of progressive dyspnea with a positive history of raising birds. 3**A** Axial chest CT (lung window) showing “head-cheese sign” (red colored squares) and fine septal thickening (blue colored arrow). 3**B** Axial chest CT (lung window) showing faint centrilobular ground glass nodules (example: orange colored square). Diagnosis: Proved patient with mixed sub-acute and chronic hypersensitivity pneumonitis (HP)

The honeycombing bronchiectasis proved to be relevant to UIP/IPF (100% prevalence and *P*-value < 0.001) (Fig. 2). The basal traction bronchiectasis and bronchiolectasis were relevant to fibrosing NSIP (100% prevalence and *P*-value < 0.001) (Fig. 2). The "head cheese" sign proved to be significant to chronic hypersensitivity pneumonitis (100% prevalence and *P*-value < 0.001) (Fig. 2).

**Fig. 3:**
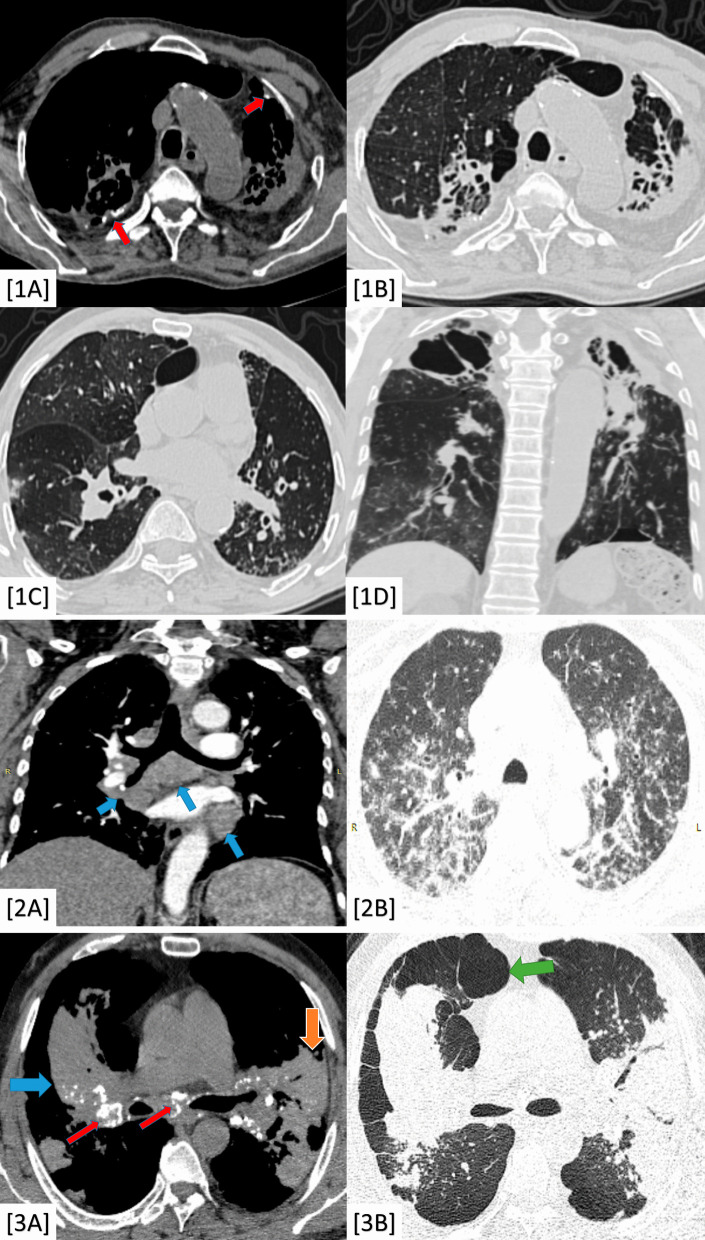
1**A**–**D** A 64-year-old male patient complaining of progressive dyspnea and productive cough. 1**A** Axial chest CT (mediastinal window) and (1**B**) Axial chest CT (lung window); both show apical fibro-cystic changes with sub-pleural cystic/emphysematous changes, tiny calcific changes (red colored arrows) and diminished left lung volume. 1**C** Axial chest CT (lung window) and (1**B**) Coronal chest CT (lung window); both showing bilateral air trapping (more on the left side) with left-sided bronchial wall thickening and tiny tree-in-bud nodules. Diagnosis: Proved patient with post-TB fibrosis with reactivation. 2**A**, **B** A 34-year-old female patient complained of progressive dyspnea and restrictive PFT. 2**A** Coronal chest CT (mediastinal window) showing mediastinal and hilar lymphadenopathy (2**B**) Axial chest CT (lung window) showing bilateral peri-bronchial thickening as well as centrilobular nodules and mixed axial (peri-bronchial) and peripheral (pleural based) peri-lymphatic nodules. Diagnosis: Proved patient with fibrosing sarcoid disease. 3**A**, **B** A 50-year-old male patient working in the glass industry (positive silica exposure) with progressive dyspnea and restrictive pulmonary functions. 3**A** Axial chest CT (mediastinal window) showing central (blue colored arrow) and sub-pleural (orange colored arrow) large consolidative patches with stippled calcific changes (red colored arrows). 3**B** Axial chest CT (lung window) showing parenchymal lung distortion with scattered nodules and sub-pleural cystic/emphysematous changes (green colored arrow). Diagnosis: Proved patient with silicosis and progressive massive fibrosis (PMF)

The presence of fibro-consolidative changes was relevant to post-TB fibrosis, sarcoidosis, PMF, and PPFE (98–100% prevalence and *P*-value < 0.001). The presence of calcific changes was also relevant to pulmonary TB and PMF on top of silicosis (100% prevalence and *P*-value < 0.001) (Fig. 3). The cavitary changes and tree-in-bud nodules were significant to pulmonary TB (24% prevalence and *P*-value < 0.001) (Fig. 3). The axial and peripheral peri-lymphatic nodules were significant to fibrosing sarcoidosis (100% prevalence and *P*-value < 0.001) (Fig. 3).

The presence of significant mediastinal and hilar nodal enlargement was relevant to post-TB fibrosis, sarcoidosis, and post-irradiation fibrosis (100%, 94%, and 52% prevalence respectively, and *P*-value < 0.001).

**Fig. 4:**
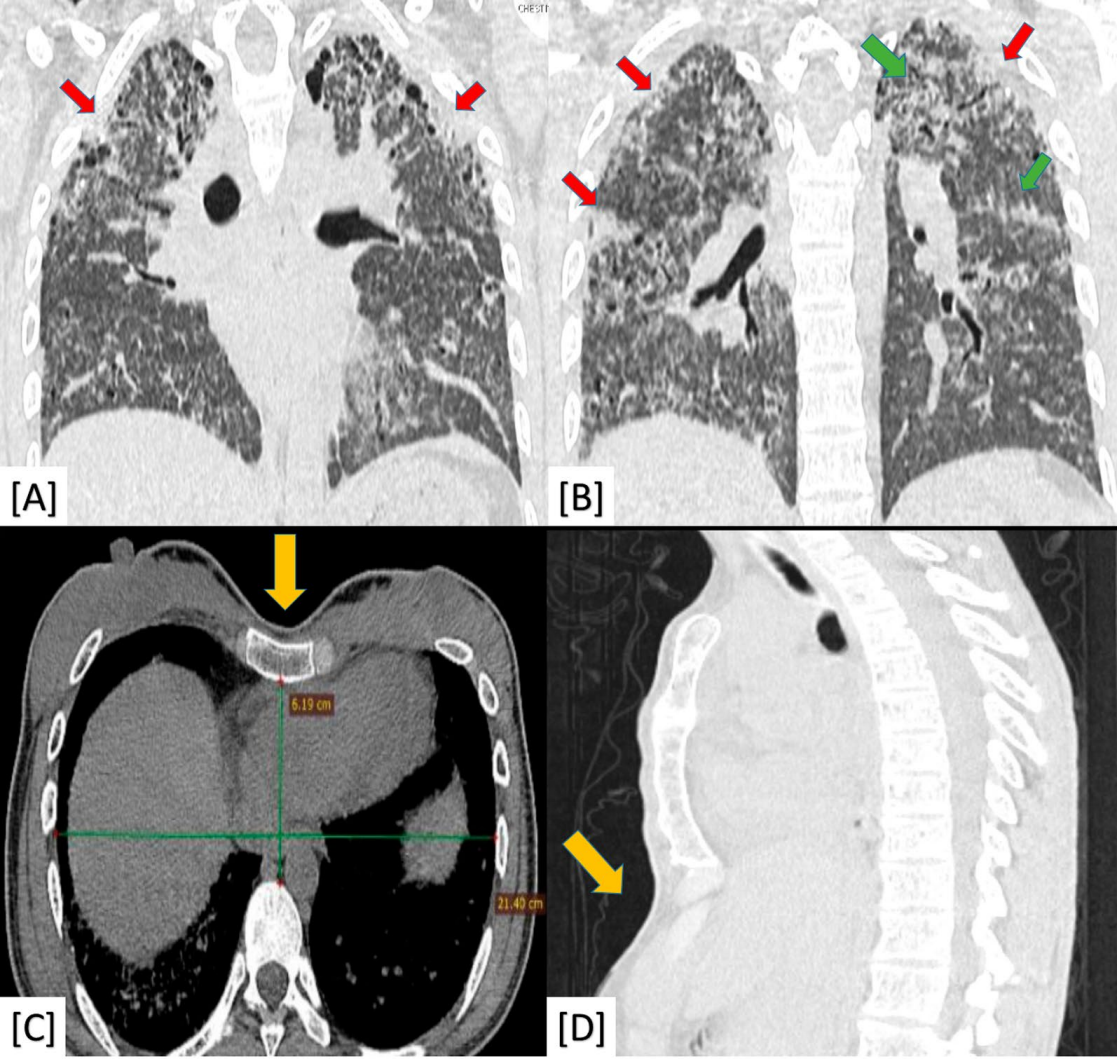
A 30-year-old female patient complaining of rapid progressive dyspnea. **A**, **B** Coronal chest CT (lung window) showing bilateral irregular pleural based (red arrows) and underlying parenchymal (green arrows) fibro-consolidative changes with sub-pleural cystic changes. **C** Axial chest CT (mediastinal window) showed a flat anterior chest wall ("platy-thorax" sign) with an evident reduction in AP diameter compared to transverse diameter. **D** Sagittal chest CT (lung window) showed also ("platy-thorax" sign). Diagnosis: Proved patient with pleuroparenchymal fibroelastosis (PPFE)

The "platy-thorax" CT-sign was significant to PPFE (100% prevalence and *P*-value < 0.001) (Fig. 4).

MRI examinations were conducted on 30/150 (20%) patients including 9/30 patients with suspicious TB scarring for scar carcinoma among a total of 21 patients proved with post-TB fibrosis and 21/30 patients with a history of malignancy and irradiation among a total of 31 patients proved with post-irradiation fibrosis.

PET/CT examinations were conducted on 40/150 (26.7%) patients including 9/40 patients with suspicious TB scarring for scar carcinoma among a total of 21 patients proved with post-TB fibrosis and 31/40 patients with a history of malignancy and irradiation in total 31 patients proved with post-irradiation fibrosis.

**Fig. 5:**
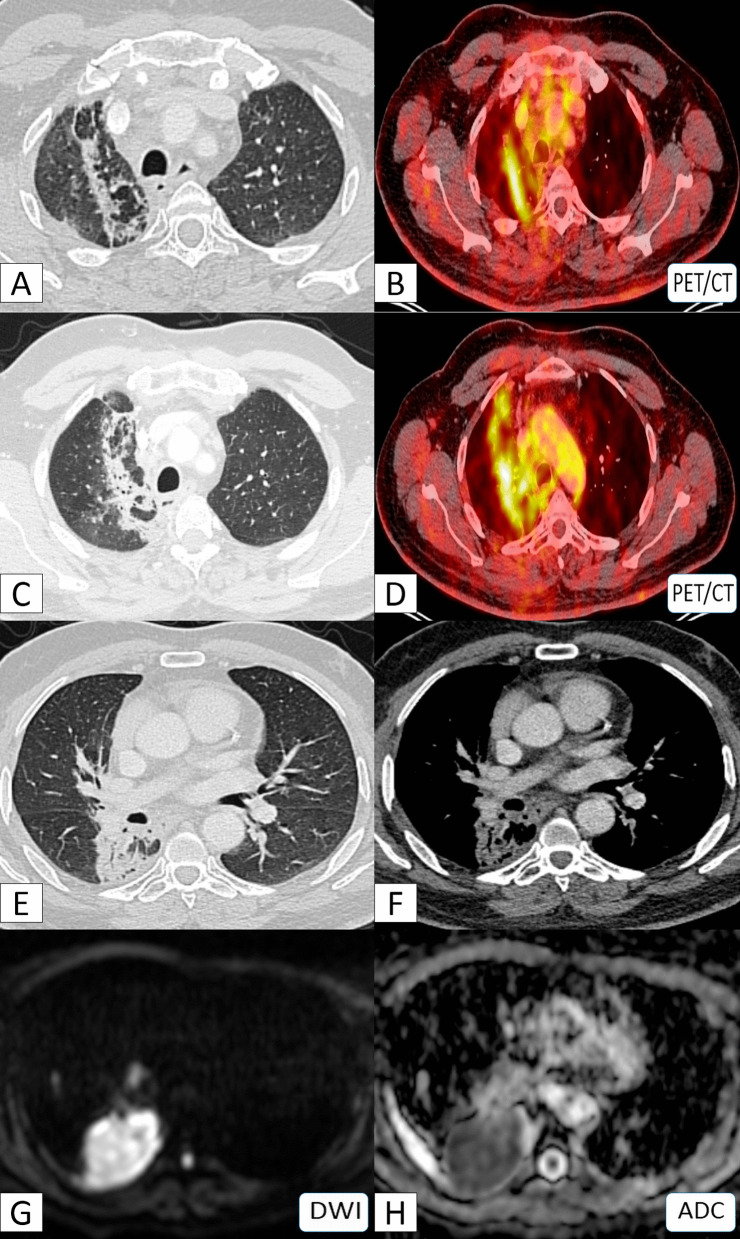
A 51-year-old male patient with a history of peripheral bronchial carcinoma in the medial aspect of the apical segment of the right lower lobe which was managed by CRT. **A**, **C** Axial chest CT (lung window) showed right apical lung scarring along the cone bead of irradiation. **B**, **D** PET/CT images showed high uptake (hot areas) at the scarring tissue. **E**, **F** Axial chest CT (lung window) showed peripheral lung carcinoma. **G** DWI showed diffusion restriction (bright signal) and **H** ADC mapping images showed corresponding low ADC value. Diagnosis: Proved patient with secondary malignancy on top of post-irradiation scarring

Lung fibrosis expressed hypo-T1 and T2 intense signals. Meanwhile, the bright T2 intense signal and DWI restriction in MRI as well as the high SUV (> 3) in PET-CT were significant for malignancy with either scar carcinoma or metastatic process (100% prevalence and *P*-value < 0.001) (Fig. 5).

**Fig. 6:**
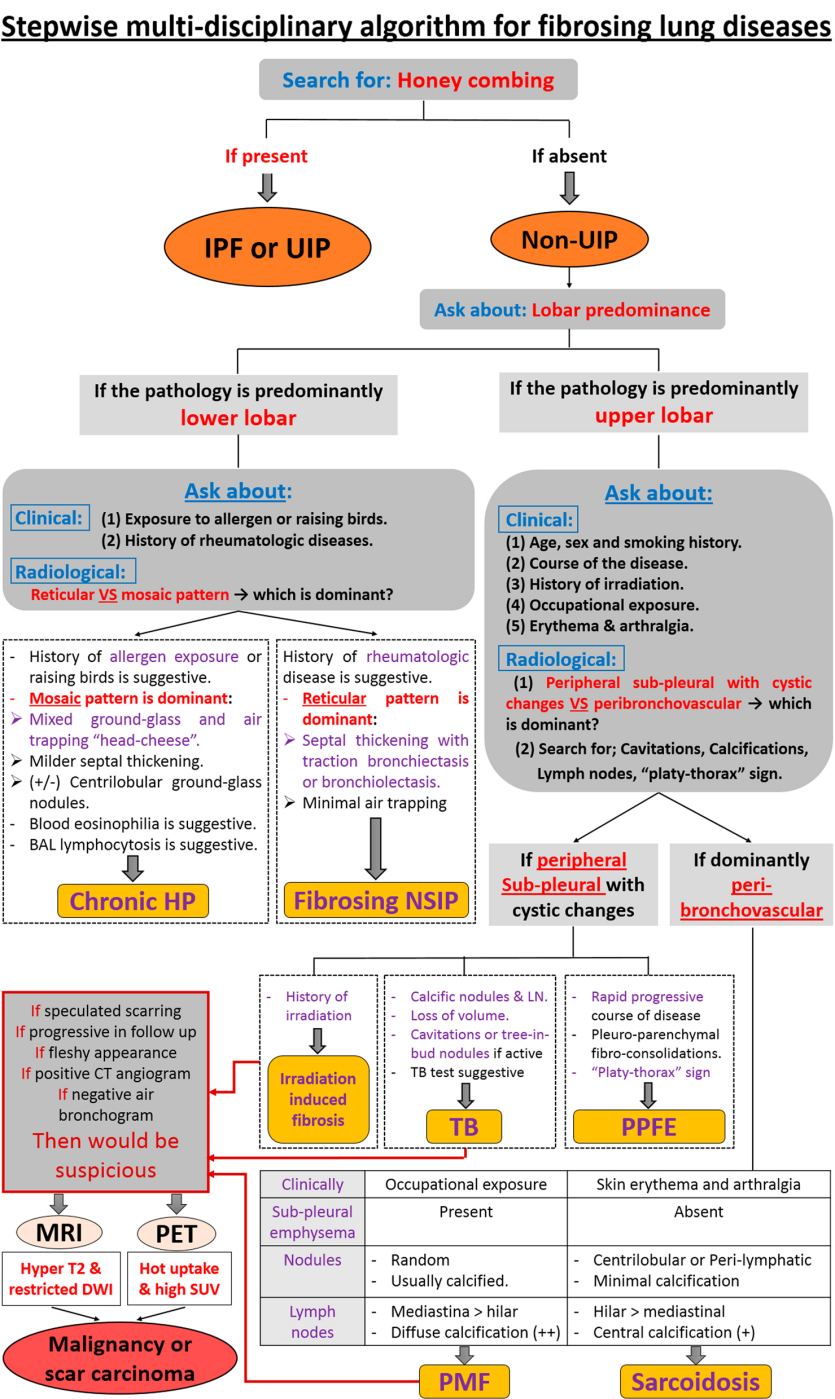
Proposed stepwise multi-disciplinary algorithm for diagnosis of fibrosing lung diseases

Based on the above-mentioned results, a stepwise multi-disciplinary algorithm was created for differentiation between fibrosing lung diseases (Fig. 6).

### Second step (validation analysis for the proposed algorithm):

Table 2 is summarizing the second step results.

**Table 2:** Statistical validation analysis for the proposed multi-disciplinary algorithm

Group	TP	FP	TN	FN	Prevalence	Sensitivity		Specificity		PPV	NPV	Accuracy	Balanced accuracy
						Value	95% CI	Value	95% CI				
*Interstitial fibrosing diseases*:													
IPF	23	0	77	0	23%	100%	85.18% to 100.00%	100%	95.32% to 100.00%	100%	100%	100%	1
Fibrosing NSIP	18	3	78	1	19%	94.7%	73.97% to 99.87%	96.3%	89.56% to 99.23%	85.7%	98.7%	96.00%	0.955
Chronic HP	15	1	81	3	18%	83.3%	58.58% to 96.42%	98.8%	93.39% to 99.97%	93.8%	96.4%	96.00%	0.91
PPFE	0	2	98	0	0%	NA	NA	98.0%	92.96% to 99.76%	NA	100%	NA	NA
*Non-interstitial fibrosing diseases*													
Post-TB fibrosis	15	0	83	2	17%	88.2%	63.56% to 98.54%	100%	95.65% to 100.00%	100%	97.7%	98.00%	0.94
Post-irradiation fibrosis	14	0	86	0	14%	100%	75.29% to 100.00%	100%	95.85% to 100.00%	100%	100%	100%	1
Fibrosing sarcoidosis	6	0	94	0	6%	100%	54.07% to 100.00%	100%	96.15% to 100.00%	100%	100%	100%	1
PMF	3	0	97	0	3%	100%	29.24% to 100.00%	100%	96.27% to 100.00%	100%	100%	100%	1
*Complications of lung fibrosis*													
Malignancy	2	0	98	0	2%	100%	15.81% to 100.00%	100%	96.31% to 100.00%	100%	100%	100%	1

*TP* True positive, *FP* False positive, *TN* True negative, *FN* False negative, *CI* Confidence interval, *PPV* Positive predictive value, and *NPV* Negative predictive value

1. The clinical records

The included 100 patients were 66 males and 34 females. Their age ranged from 41 to 73 years (mean age 59.1 ± 9.2 SD)**.**

2. The type and complications of fibrosing lung disease

**Fig. 7:**
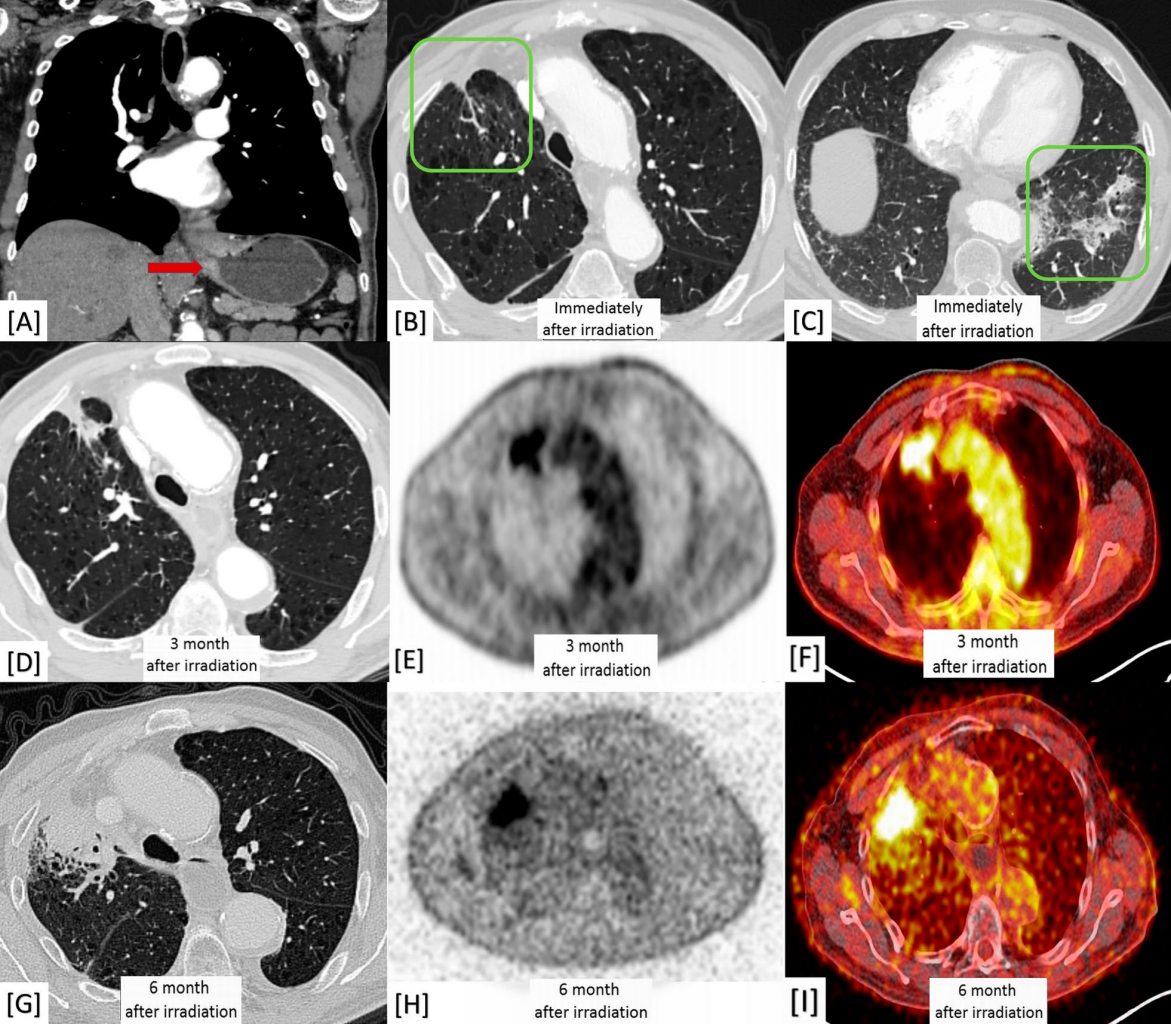
A 63-year-old male patient with a history of gastro-esophageal carcinoma managed by CRT. **A** Pre-management coronal chest CT (mediastinal window) expressing the gastro-esophageal malignant mural thickening (red arrow). **B** Axial chest CT (lung window) immediately after radiotherapy cycles showing lung emphysematous changes with right anterior upper lobar small scar showing speculated outlines (green square). **C** Axial chest CT (lung window) immediately after radiotherapy cycles showing left basal parenchymal scarring with ground-glass halo (green square). **D** Three-month post-irradiation axial chest CT (lung window) showing progression in size and peripheral speculations of the scar. **E**, **F** Three-month post-irradiation PET/CT scans showing high FDG-uptake. **G** Six-month post-irradiation axial chest CT (lung window) showing further progression in size and peripheral speculations of the scar with fleshy soft tissue appearance with interrupted air bronchogram. **H**, **I** Six-month post-irradiation PET/CT scans confirming size progression and showing higher FDG-uptake and higher SUV. Diagnosis: Proved patient with metastatic deposit

**Fig. 8:**
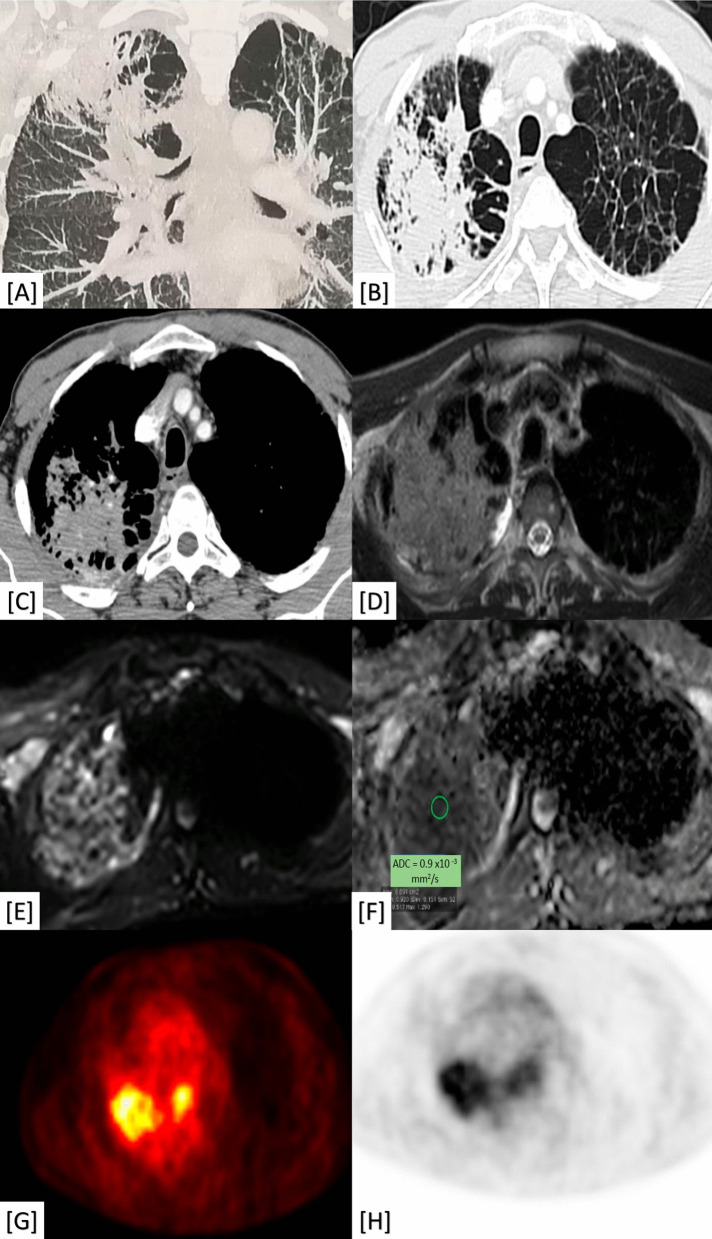
A 58-year-old male heavy smoker patient with a history of old TB complaining of progressive dyspnea. **A** Initial coronal CT (lung window) showing apical lung scarring. **B** Follow-up chest CT (lung window) showed progression in size of the scar. **C** Follow-up chest contrast-enhanced CT (mediastinal window) showing fleshy soft tissue appearance with internal CT angiogram and no air bronchogram. **D** T2WI MRI revealed a hyper-intense signal of the tumor. **E** DWI-MRI revealed restriction diffusion. **F** ADC mapping revealed Low ADC value (0.9 × 10^−3^ mm^2^/s). **G**, **H** PET-CT scans showing high FDG uptake. Diagnosis: Proved patient with scar carcinoma sequel to old TB-fibrosis

Chronic fibrosing IIPs were encountered in (60/100) patients, including UIP/IPF (23%), fibrosing NSIP (19%), and chronic hypersensitivity pneumonitis (18%). Non-interstitial fibrosing lung diseases were less common (40%), including post-TB fibrosis (17%), post-irradiation pneumonitis (14%), sarcoid disease (6%) and progressive massive fibrosis on top of pneumoconiosis (3%). Malignant changes were detected in two patients; one of them proved with scar carcinoma on top of post-TB fibrosis and COPD (Fig. 7), while the other proved with post-irradiation metastatic spread (Fig. 8).

The application of the proposed algorithm succeeded in the diagnosis of 94/100 patients (true positive cases), but it failed to diagnose the other six patients. Three patients who were proved with chronic hypersensitivity pneumonitis were falsely diagnosed with fibrosing NSIP. A single patient who was proven with fibrosing NSIP was falsely diagnosed with chronic HP. Two patients proved with post-Tb fibrosis were falsely diagnosed as PPFE.

3. The validation analysis statistical results

The application of the proposed algorithm proved to be valid for the diagnosis of fibrosing lung diseases after reaching 83.3%–100% sensitivity, 96.3%–100% specificity, 85.7%–100% positive predictive value (PPV), 96.4%–100% negative predictive value (NPV), and 96%–100% accuracy, with balanced accuracy = 0.91–1.

## Discussion

Unlike the surgical biopsy which examines only small tissue fragments, MDCT examines the lung entirely [14]. The new guidelines also stated that MDCT can diagnose UIP/IPF without the need for tissue biopsy confirmation by applying a new categorization based on MDCT findings into; UIP pattern, possible UIP pattern, and consistent with UIP pattern [14]. But because of the overlapping CT signs, the dilemma of the diagnosis of non-UIP diseases is still confusing. Therefore, communication between pulmonologists, radiologists, and pathologists is mandatory to yield more specific diagnoses avoiding the hazards of lung biopsy as much as possible [15–19].

In this study, the authors retrospectively studied the prevalence and significance of the major clinico-laboratory and radiological parameters that contribute to the diagnosis of fibrosing lung diseases among 150 patients. Then, based on the available results, they proposed a stepwise multi-disciplinary algorithm combining these data. Eventually, they prospectively applied this algorithm to 100 patients to test its validity.

This study matched Edey et al. [20] and Nayak et al. [21] who emphasized the importance of the characteristic basal sub-pleural honeycombing bronchiectasis in the diagnosis of UIP/IPF.

Keeping with Hodnett et al. [22], the traction bronchiectasis and bronchiolectasis in this study were significant to fibrosing NSIP side by side to common basal sub-pleural ground-glass attenuation. Additionally and contrary to chronic HP, the peri-bronchial involvement proved in this study to be a non-specific finding. This matches Sundaram et al. [23].

Furthermore, this study agrees with Lynch et al. [24] regarding the importance of a positive history of allergen exposure and the "head cheese" CT sign which reflects obliterative bronchiolitis in the diagnosis of hypersensitivity pneumonitis.

Similar to Portillo et al. [5], the peripheral pleuro-parenchymal fibro-consolidative changes with parenchymal distortion and "platy-thorax," in patients with a rapidly progressive course of dyspnea, were diagnostic for PPFE.

Additional to Abehsera et-al. [9], who stated that fibrosing sarcoidosis typically affects the upper and middle lung zones with parenchymal distortion and peri-lymphatic nodules, this study proved the significance of the peri-bronchovascular distribution and clarified the importance of skin erythema and arthralgia as relevant clinical findings. This can differentiate fibrosing sarcoidosis from PMF.

Confirming Begin et al. [25], PMF complicated pneumoconiosis in patients with positive industrial exposure was characterized by upper lobar peri-broncho-vascular involvement fibro-consolidative changes with distal air trapping and sub-pleural emphysema. Silicosis was the most common underlying pneumoconiosis with calcific changes, calcific nodules, and calcific mediastinal nodes.

The peripheral sub-pleural involvement can differentiate post-TB fibrosis from PMF, in addition to tree-in-bud nodules or cavitary changes if present. This is matching Kim et al. [8] who described the thoracic sequelae and complications of tuberculosis.

As mentioned in Wennberg et al. [26], the irradiation fibrosing pneumonitis in this study was characteristically confined to the field of irradiation beam in patients with lung, breast, and thyroid cancer.

This study collaborated the rule of MRI and PET-CT in the detection of malignant changes that complicate some fibrosing lung diseases such as post-TB fibrosis (scar carcinoma) and post-irradiation malignancy. Eventually, it was found that the bright T2 signal together with diffusion restriction in MRI and high SUV in PET/CT were significant diagnostic parameters. This agrees with Razek et al. [27], and Usuda et al. [28], also it can add to Antoch et al. [29] who discussed the role of combined PET/MRI in the detection of malignant changes in tissue scarring.

The main merits of this study over the previous literature were:

1. The collaboration of the clinico-laboratory, MDCT, MRI, and PET/CT data in a single stepwise algorithm after statistical analysis of their significance.
2. The other important advantage of this study was the step of validation analysis.

This study faced four limitations;

1. The first limitation was regarding the number of the included patients despite the long time interval of the study which approximated six years; Only 150 patients were included in the first step because of the absent pathologic proof for many other patients who were excluded from the study, this can be explained by the universal concept of limiting unnecessary lung biopsy. Also, only 100 patients were available to be included in the second step of the study during the COVID-19 pandemic restrictions.
2. The second limitation was the relatively limited distinguishable power of the algorithm for differentiation between PPFE and post-TB fibrosis.
3. The third limitation was the absence of secondary UIP cases of upper lobar honeycombing bronchiectasis; however, these patients could be easily distinguished based on the first step of the algorithm.
4. The fourth limitation was the absence of MRI or PET/CT examinations for patients with PMF because of the rare incidence of malignant transformation. Still, the main concept of malignant significant predictors proposed by the algorithm can distinguish these rare cases if present

Because of these limitations, we recommend further large group validation testing of this proposed algorithm whenever possible.

## Conclusions

A valid stepwise multi-disciplinary algorithm was proposed for the diagnosis of interstitial and non-interstitial fibrosing lung diseases to limit the need and hazards of lung biopsy. It contributed significant clinico-laboratory data, MDCT features, T2-WI and DWI-MRI findings as well as PET/CT results.

## Data Availability

The datasets used and/or analyzed during the current study are available from the corresponding author on reasonable request.

## Abbreviations

BAL: Broncho-alveolar lavage
DWI: Diffusion weighted image
HP: Hypersensitivity pneumonitis
IIPs: Idiopathic interstitial pneumonias
IPF: Idiopathic pulmonary fibrosis
MDCT: Multi-detector computed tomography
MRI: Magnetic resonance imaging
NPV: Negative predictive value
NSIP: Non-specific interstitial pneumonia
PET/CT: Positron emission tomography
PMF: Progressive mass fibrosis
PPFE: Pleuroparenchymal fibroelastosis
PPV: Positive predictive value
TB: Tuberculosis
SUV: Standardized uptake value
UIP: Usual interstitial pneumonia
VATS: Video-assisted thoracoscopic surgery
